# Tumour heterogeneity promotes collective invasion and cancer metastatic dissemination

**DOI:** 10.1098/rsos.161007

**Published:** 2017-08-23

**Authors:** Adrien Hallou, Joel Jennings, Alexandre J. Kabla

**Affiliations:** Department of Engineering, University of Cambridge, Cambridge CB2 1PZ, UK

**Keywords:** cancer, tumour heterogeneity, metastasis, collective invasion

## Abstract

Heterogeneity within tumour cell populations is commonly observed in most cancers. However, its impact on metastatic dissemination, one of the primary determinants of the disease prognosis, remains poorly understood. Working with a simplified numerical model of tumour spheroids, we investigated the impact of mechanical heterogeneity on the onset of tumour invasion into surrounding tissues. Our work establishes a positive link between tumour heterogeneity and metastatic dissemination, and recapitulates a number of invasion patterns identified *in vivo*, such as multicellular finger-like protrusions. Two complementary mechanisms are at play in heterogeneous tumours. A small proportion of stronger cells are able to initiate and lead the escape of cells, while collective effects in the bulk of the tumour provide the coordination required to sustain the invasive process through multicellular streaming. This suggests that the multicellular dynamics observed during metastasis is a generic feature of mechanically heterogeneous cell populations and might rely on a limited and generic set of attributes.

## 1. Introduction

Metastasis, the process during which cancer cells migrate away from a primary tumour and disseminate in other organs, accounts for more than 90% of cancer fatalities [1]. At the cellular level, malignant cell behaviour has been associated with an accumulation of gene mutations and a malfunction of key regulatory signalling pathways [2,3] such as TGF-β or Twist [4,5]. Moreover, following earlier pioneering works [6], recent analyses have established that most solid tumours are not homogeneous but a mosaic of various cell populations with different genetic and phenotypic traits [7]. Owing to the variability in these factors across different pathologies, patients and even tumours, no unique mechanistic pathway leading to the induction and progression of metastasis has been yet characterized [8,9].

By contrast, recent advances in imaging techniques at the tissue scale [10–13], along with histopathological studies on patients’ biopsies [13], draw a surprisingly unified picture of cancer invasion in terms of cell migration behaviours. In most cancers, despite tremendous variations in gene expression and biochemical environment, tumour cells are able to invade collectively by maintaining intercellular coordination [13–18], generating multicellular structures such as cell sheets, strands or clusters remaining cohesive and polarized [14]. The accumulated knowledge about collective cell migration [19] and its quantitative biophysical models [20,21] is therefore likely to inform the question of tumour invasion at a system level. Recent results have also highlighted a link between tumour heterogeneity and metastatic dissemination through collective invasion. Experimental evidence suggests that non-invasive or poorly invasive tumour cells can be driven to invade by invasion-competent cancer cells [17,18,22–24] or stromal fibroblasts [15,16,25] through a ‘leader/follower’ invasion mechanism [13,26].

In this article, we aim to address the question of the impact of tumour heterogeneity on malignancy. We use a minimal model for collective cell migration in order to identify both the individual and synergistic effects of cell coordination and cell heterogeneity on tumour invasion.

## 2. Modelling approach

Our model involves a Cellular Potts algorithm [27] including a self-propelled term to account for the active motion of cells [28–30]. It builds on the computational framework introduced previously [30] to study the collective dynamics of cell populations invading two-dimensional resistive environments, most often at the planar interface between two different tissues, along tracks created by blood vessels, myofibres or nerve bundles [12,31].

This approach is appealing, as the Cellular Potts algorithm has already been used to model a variety of tissue behaviours associated with cancer invasion [32–34] or heterogeneous collective migration during angiogenesis [35,36]. Furthermore, our model [30] has been already fully characterized in the context of homogeneous motile cell populations, and has made numerous predictions verified in experiments [37–39]. Details of the computational method and numerical values of the parameters used in *in silico* experiments are provided in the electronic supplementary material.

The key physical parameters of the model are the cell size (or area in two dimensions), its compressibility (low compared with other forces at play), the surface energy of the cell interface which controls the shear stiffness, tissue cohesion and some aspects of energy dissipation. In addition, each motile cell *i* exerts a motile force *μ*_*i*_***n***_*i*_ acting on a planar substrate, where ***n***_*i*_(*t*) defines the direction of its front–rear polarity, and *μ*_*i*_ its active motile force magnitude. The polarization vector ***n***_*i*_(*t*) also evolves over time and is set along the direction of the mean previous displacements of the cell in the time interval [*t*−*τ*,*t*]. It represents the time-scale at which cell polarity responds to external stimuli and controls the persistence length of the cell trajectory in the absence of other cells [28,30]. Simulating the evolution of the system relies on a stochastic Monte-Carlo algorithm (cf. electronic supplementary material), which defines the arbitrary time unit used in this paper, the Monte Carlo Step (MCS). The associated noise or stochasticity level is also a parameter, although it has been found not to play a critical role in the range of surface energy and motile force explored here [30].

Earlier numerical work on migratory cell populations [30] has evidenced two behavioural transitions in cell dynamics (cf. electronic supplementary material, table S1). Keeping all parameters but the motile force *μ* constant, the system exhibits: (i) a critical force *μ*_s_ needed for a single cell to migrate through a non-motile cell population; (ii) in homogeneous cell populations, a transition from static epithelial behaviour to collective streaming, at a critical value of the motile force parameter *μ*_c_<*μ*_s_; and (iii) a regime of motile forces *μ*_c_<*μ*<*μ*_s_ associated with large spatial and temporal correlations of collective cell behaviours. The latter is of particular interest when considering a minimal tumour model, composed of a two-dimensional spheroid of motile cells surrounded by cohesive tissues that mechanically resist invasion as often observed *in vivo* [12].

**Figure 1.**
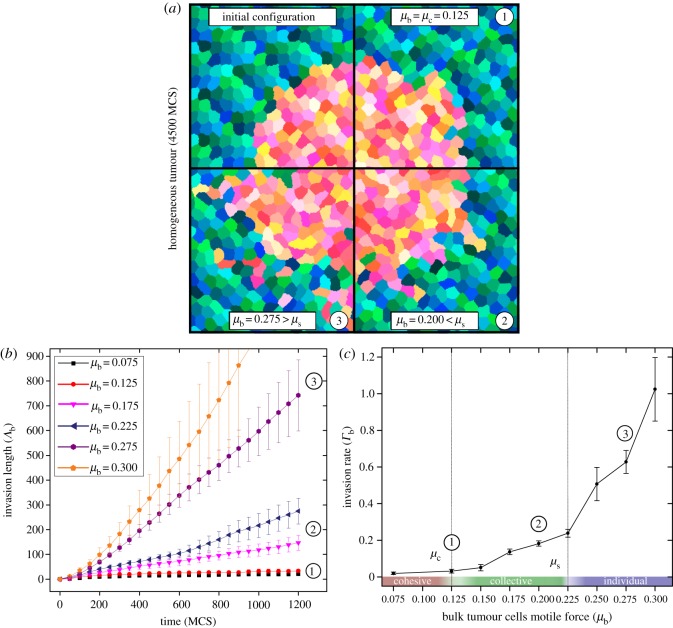
Collective invasion for homogeneous tumours. (*a*) Snapshots of *in silico* experiments. Top-left quadrant represents the initial state of the system (green–blue cells are non-motile, yellow–red cells are bulk tumour cells). The three other quadrants (1–3) represent the state of the system for different values of *μ*_b_ at *t*=4500 MCS. (*b*) Invasion length of bulk tumour cells *Λ*_b_ with respect to time, for different values of *μ*_b_. (*c*) Invasion rate *Γ*_b_ with respect to *μ*_b_. Details on the computation of plotted quantities from *in silico* experiments are provided in the electronic supplementary material. *μ*_c_ and *μ*_s_ values relate to cell migration behaviour transitions. Error bars are the standard error of the mean (s.e.m.).

As introduced previously [30], homogeneous tumour spheroids here consist of bulk tumour cells of motile force *μ*_b_ surrounded by a population of non-motile cells, all other parameters of the model being kept uniform in order to avoid biasing cell migration towards surrounding tissues. Cells displayed behaviours ranging from no invasion, *μ*_b_<*μ*_c_, to widespread single cell dispersal for *μ*_b_>*μ*_s_ (cf. figure 1*a*; electronic supplementary material, movies S1, S2, S3, S4 and S5). The regime where *μ*_c_<*μ*_b_<*μ*_s_ is remarkable. Invasion of tumour cells, although slow, is observed and takes the form of finger-like collective patterns (cf. figure 1*a* bottom-right quadrant; electronic supplementary material, movie S2), as described in many *in vivo* studies [10,11,13]. This indicates that although no individual cell is able to invade in this regime, collective effects occasionally lead to larger mechanical forces sufficient to enable invasion. While this might capture a fundamental mechanism involved in the early stages of collective invasion, finger formation, in these *in silico* experiments, is a rare event and might not account for highly malignant situations observed *in vivo*. Several reasons may explain this limited amount of collective invasion. The strength of collective invasion [17,40] tends for instance to be reinforced *in vivo* by complementary biochemical processes such as enzymatic tissue remodelling [40], a fact already reproduced *in silico* with some success [29]. The analysis presented below demonstrates how mechanical heterogeneities may also dramatically increase the dynamics of cancer invasion. The homogeneous case is further analysed to develop a control dataset from which the effects due to a small sub-population of mechanically stronger cells can be assessed. The model will specifically focus on the onset of invasion, a regime where cell motility is the key driver [22]. We therefore assume that cell growth and proliferation effects can be ignored.

## 3. Results and discussion

### 3.1. Invasion from homogeneous tumours

In all numerical experiments, we selected an initial tumour radius *r*_0_ of the order of 10 cell diameters, corresponding to a population of around 300 cells (figure 1*a*), which is a typical size observed in similar *in vivo* [22] and *in vitro* experiments [17]. The extent of invasion is characterized by the length *Λ*_b_(*μ*_b_,*t*) corresponding to the sum of the distances from the initial tumour boundary to each cancer cell *i* that is outside the initial boundary at time *t* [30]. This cumulative quantity encompasses both the spatial extent of invasion and the number of invading cells. *Λ*_b_ varies almost linearly with time for the first thousand MCS, as shown in figure 1*b* in the context of a homogeneous population of motile cells. The capacity of bulk tumour cells to invade surrounding tissues can, therefore, be characterized as a rate of invasion at short time scales, *Γ*_b_(*μ*_b_)=*Λ*_b_(*μ*_b_,Δ*t*)/Δ*t*. The evolution of *Γ*_b_ as a function of the cells’ motile force *μ*_b_ is represented in figure 1*c*, for Δ*t*=1200 MCS. The sensitivity and convexity of the invasion rate to the motile force of the tumour cells is a preliminary indication that heterogeneities in the motile properties of the cancer cells could have a significant effect on invasion rates and patterns.

### 3.2. Invasion from heterogeneous tumours

**Figure 2.**
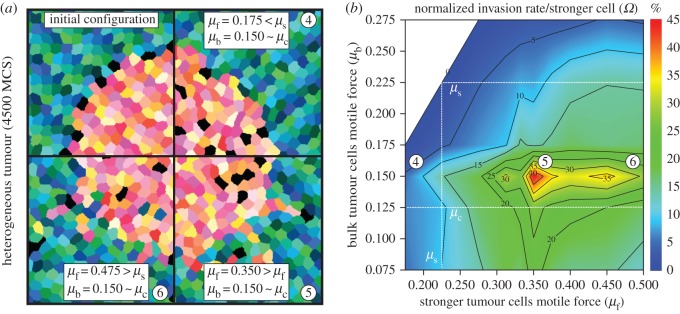
Collective invasion for heterogeneous tumours. (*a*) Snapshots of *in silico* experiments with 24 stronger cells. Top-left quadrant represents the initial state of the system (green–blue cells are non-motile, yellow–red cells are bulk tumour cells, black cells are stronger tumour cells). The three other quadrants (4–6) represent the states of the system for different values of *μ*_b_ and *μ*_f_ at *t*=4500 MCS. (*b*) Heat map of the normalized invasion rate per stronger cell *Ω*. The maximum is observed for *μ*_b_=0.150 and *μ*_f_=0.350. Details on the computation of *Ω* from *in silico* experimental data are provided in the electronic supplementary material.

Heterogeneity is introduced in the system in the form of a small number, *N*_f_, of cells positioned at the boundary of the tumour (cf. figure 2*a*), with a larger motile force, *μ*_f_. This configuration is commonly found *in vivo* [17] and provides a reproducible starting point to study the initiation of the invasion process. We analysed the behaviour of the system for *N*_f_= 6, 12 or 24, i.e. about 2%, 4% or 8% of the total motile cell population respectively, proportions again consistent with experimental observations [15–18]. As for the homogeneous tumour case, the invasive behaviour of bulk tumour cells in the presence of these particular cells is monitored by computing the invasion length *Λ*_f_(*μ*_b_,*μ*_f_,*N*_f_,*t*) and the corresponding invasion rate *Γ*_f_(*μ*_b_,*μ*_f_,*N*_f_)=*Λ*_f_(*μ*_b_,*μ*_f_,*N*_f_,Δ*t*)/Δ*t*.

The effect of heterogeneity on the invasion efficiency is measured as the percentage increase in the invasion rate per stronger cell: *Ω*(*μ*_b_,*μ*_f_)=(∂_*N*_f__*Γ*_f_)/*Γ*_b_ (figure 2*b*). As expected, the presence of stronger cells brings no significant enhancement to the invasion efficiency for *μ*_b_≥*μ*_s_, where the bulk of the cancer cells are able to invade on their own. Furthermore, when both bulk and stronger tumour cells generate a motile force below the threshold for single cell invasion, there is only a marginal effect on invasion (cf. electronic supplementary material, movie S6).

By contrast, when the bulk of the tumour is in the regime of collective invasion (*μ*_c_≤*μ*_b_≤*μ*_s_), the addition of a small number of stronger cells (*μ*_f_≥*μ*_s_) has a dramatic effect. Each of them significantly increases the invasion rate (20%≤*Ω*≤45%), with an optimum for *μ*_b_≈0.150 and *μ*_f_≈0.350.

### 3.3. Morphological analysis

To understand the origin of this optimum, we analysed the morphology of the tumour at the onset of invasion. Figure 2*a* illustrates the typical spatial arrangements between stronger and bulk tumour cells in protrusive fingers, in which stronger cells are commonly seen initiating and leading fingers (cf. electronic supplementary material, movie S7). To quantify these morphological features, a graph-based method is used to identify the contact network of cells leaving the tumour. Fingers are defined as groups connected to the tumour body that are more than two cells away from the tumour boundary. The tip is defined as the cell of the finger which is topologically the furthest away from the original tumour boundary. The length of a finger is the length of the shortest path between its tip and the original tumour boundary, measured in number of cells.

**Figure 3.**
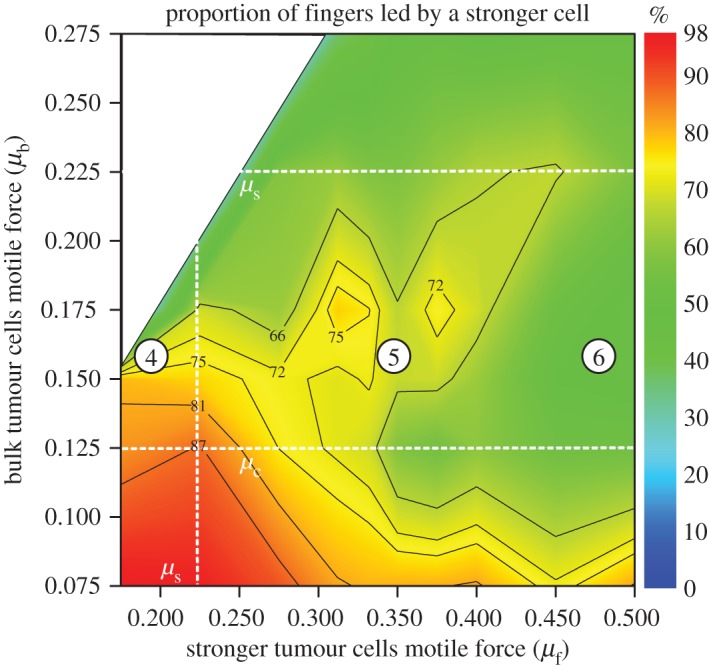
Morphology of invading structures in heterogeneous tumours. Heat map of the proportion of fingers led by a stronger cell. Details on the computation of this quantity from *in silico* experiments data are provided in the electronic supplementary material. The dataset presented is the same as for figure 2*b*.

Figure 3 shows the proportion of fingers led by a stronger cell, pooling all data from experiments with 6, 12 and 24 stronger cells. In the regime of collective invasion (*μ*_c_≤*μ*_b_≤*μ*_s_), up to 70% of the fingers can be led by stronger cells, far more than what would be expected from their density at the tumour boundary (approx. 25–30%) in average. Stronger cells, therefore, promote invasion by acting as leader cells.

The trend observed in figure 3 along the *μ*_b_ axis is expected. When *μ*_b_>*μ*_s_, all cells can invade and fingers are equally likely to be led by any cell type; consequently, the proportion of fingers led by a stronger cell decreases. Similarly, when *μ*_b_<*μ*_c_, bulk cells cannot migrate on their own and any of the rare emerging invading structures would be pulled by stronger cells. The fact that the proportion of fingers led by a stronger cell decreases with *μ*_f_ for large values of *μ*_f_ is more surprising (cf. region between points 5 and 6 in figure 3). Why would an increase in the motility of the stronger cells reduce their ability to lead fingers?

**Figure 4.**
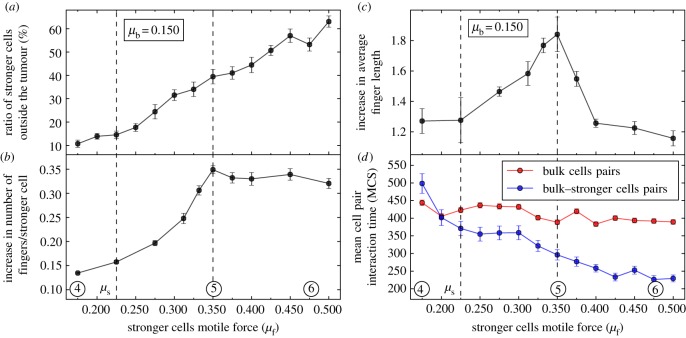
Impact of tumour heterogeneity on the dynamics of invading structures. (*a*) Proportion of stronger cells outside the tumour as a function of *μ*_f_, for *μ*_b_=0.150. (*b*) Increase in number of fingers per stronger cell as a function of *μ*_f_, for *μ*_b_=0.150. (*c*) Relative increase in average finger length with respect to the homogeneous case (*μ*_b_=0.150) as a function of *μ*_f_. (*d*) Mean contact time for both bulk–bulk and bulk–stronger cells pairs as a function of *μ*_f_. Details on the computation of these quantities from *in silico* experimental data are provided in the electronic supplementary material. Circled numbers relate to results in figures 2*a* and 3. Error bars stand for s.e.m.

Figure 4*a* shows that the proportion of stronger cells leaving the tumour by a specific time (approx. 4000 MCS) increases monotonically with *μ*_f_, up to more than 60% for *μ*_f_=0.5. This is associated with an increase in the number of fingers pulled per stronger cells (figure 4*b*). In the range 0.175<*μ*_f_<0.350, this number matches the proportion of stronger cells leaving the tumour. However, the number of fingers pulled per stronger cell plateaus for *μ*_f_>0.350, suggesting a change in the interaction between bulk and stronger cells. Not only does the number of fingers saturate, but their length also diminishes as *μ*_f_ increases beyond 0.350 (figure 4*c*). The finger initiation step is, therefore, not responsible for the invasiveness decay at large *μ*_f_. To interpret this behaviour, we analysed the distributions of interaction times between motile cells during the first 2000 MCS (cf. electronic supplementary material, figures S1 and S2). Figure 4*d* shows the mean contact duration between two bulk cells, and between a bulk cell and a stronger cell, as a function of *μ*_f_. These data show that, as *μ*_f_ increases, there is a growing mismatch between these interaction times; a high motile force prevents the stronger cells from maintaining sustained contacts with bulk cells. This implies that, for large *μ*_f_, stronger cells detach from bulk cells too quickly to generate long fingers, leaving short fingers behind that are unable to invade further into the tissue (cf. electronic supplementary material, movie S8). These results shed light on the invasion rate data presented in figure 2*b* as the invasion rate is expected to scale with both the number of fingers and their length. Indeed, figure 2*b* can be approximatively replicated by multiplying the increase in number of fingers per stronger cell and relative increase in finger length (cf. electronic supplementary material, figures S4, S5 and S6). Therefore, the optimum invasion is a signature of the distinct requirements for (i) initiating finger like protrusions and (ii) sustaining their penetration into surrounding tissues.

Another observation from the model is the clustering of the invading cell population due to fingers breaking up from the primary tumour. These tumour cell clusters have a typical size of two to six cells and can be heterogeneous (cf. electronic supplementary material, figure S7). Although agreement with quantitative experimental data might be coincidental, it is worth noting that both the size and the degree of heterogeneity are close to the typical values measured for circulating tumour cell clusters *in vivo* [41–43].

## 4. Conclusion

By combining two key observations of *in vivo* cancer metastasis, collective cell behaviour and tumour phenotypic heterogeneity, the minimal model presented here demonstrates that mechanical heterogeneity has a dramatic impact on cancer invasion dynamics. The origin of such effect is not associated with a growth driven instability [44]. We anticipate, however, that growth and cell division would enhance collective invasion behaviours as has been reported [12]. In our model, the invasion rate is maximized when the population contains cells with complementary traits, combining a few invasion competent cells with a bulk population able to collectively migrate and follow leading cells away from the main tumour. Such a qualitative interpretation of the results is consistent with the fact that various forms of tumour heterogeneity lead to similar invasion patterns; stromal fibroblasts [15,16], genetic or phenotypic variability [17,18] or partial epithelial-to-mesenchymal transition [45,46] within the tumour are all able to trigger collective invasion patterns led by highly motile cells. These similarities can be explained by the fact that generic physical interactions are sufficient to reproduce leader/follower dynamics in invading finger-like multicellular protrusions. Although we considered heterogeneities in cell motile forces, we anticipate that differences in other cellular attributes such as proteolytic activity [40], adhesion [46] or cell stiffness [47] are likely to lead to similar invasion patterns when combined with collective effects in the cell population.

A general approach to the physics of heterogeneities in collective systems is still lacking. This work demonstrates that developing such a framework would represent an important contribution to understanding a number of biological processes both in animal development and pathologies.

## Supplementary Material

Supplementary Figures and Methods

## Data accessibility

The figures’ data and the code used to generate the raw data are available at: https://doi.org/10.17863/CAM.11252. Parameters used in the simulations are presented in the electronic supplementary material.
