# Supplementary material for: Tumour heterogeneity promotes collective invasion and cancer metastatic dissemination
Source: R Soc Open Sci. 2017 Aug 23;4(8):161007. doi: 10.1098/rsos.161007 (PMC5579073; doi:10.1098/rsos.161007)
Supplement: Supplementary Figures and Methods [file rsos161007supp1.pdf]

Supplementary Materials

Tumour heterogeneity promotes collective invasion and cancer metastatic dissemination

Adrien Hallou, Joel Jennings and Alexandre Kabla

Department of Engineering, University of Cambridge, Cambridge, CB2 1PZ, UK.

July 11, 2017

Modelling method

In the model, using the computational framework already introduced in [1], tissues are represented on a 2D lattice of 1280 by 1280 pixels, where the value i of each pixel codes for the identity of the cell on that location. The cell volume $V_i(t)$ is constrained to a target value V_0 by a bulk modulus κ. The interfacial cell-cell interactions, such as adhesion or cortical tensions, are all accounted for by a single parameter, J, which sets the surface energy of cell membrane. The active motion of each cell is driven by a motile force $\boldsymbol{f}_i\,(t) = \mu_i \boldsymbol{n}_i$ acting on a fixed substrate, where $\boldsymbol{n}_i\,(t)$ defines the cell polarization axis and μ_i its active motile force magnitude, which depends on the cell type. The motile force is used to calculate an instantaneous migration potential which captures the mechanical work generated by each active cell: $w_i = -\boldsymbol{f}_i \cdot \boldsymbol{r}_i$, where $\boldsymbol{r}_i$ represents the position of the cell centroid. At any given time, volume constraint, cell-cell interactions and active migration energy terms can be combined in to an overall energy function, $E(t)$:

$$E = \sum_{k,k'} J\left(1 - \delta_{i(k)i(k')}\right) + \sum_i \frac{1}{2}\kappa\left(V_i - V_0\right)^2 + \sum_i w_i \tag{1}$$

where i represents the cell index and (k, k') represent pairs of neighbouring pixels. $\delta_{i(k)i(k')}$ is 1 when both pixels belong to the same cell and 0 otherwise.

The system dynamics results from the iterative minimisation of this energy function through the Metropolis Monte-Carlo algorithm [1], where the level of noise or stochasticity in the system is accounted for by the parameter T. Time is here expressed in Monte Carlo steps (MCS), where 1 MCS corresponds to an average of one iteration per pixel over the whole lattice. The polarization vector of a cell, $\boldsymbol{n}_i(t)$, also evolves over time and is set along the direction of the cell displacements in the time interval $[t-\tau, t]$:

$$\boldsymbol{n}_i(t) = \frac{< \vec{v}_i(t) >_{[t-\tau,t]}}{|< \vec{v}_i(t) >_{[t-\tau,t]}|} \tag{2}$$

To highlight the role of cell migration, all cell populations have the same mechanical, interfacial and dynamical properties (V_0, κ, J, τ, T). They only differ in the value taken by their motile forces (μ_i).

Parameters of the model

List of the model parameters and numerical values used in *in silico* experiments.

**Name of the parameter**	**Symbol**	**Numerical Value**	**Dimension/Unit**
Cell bulk modulus	κ	1.0	$[E/L^2]$
Cell-cell interfacial energy	J	5.0	$[E/L]$
Bulk tumour cells motile force	μ_b	$[0.075, 0.275]$	$[E/L]$
Stronger tumour cells motile force	μ_f	$[0.175, 0.500]$	$[E/L]$
Cell polarity persistence time	τ	10.0	$[t]$/MCS
Noise level	T	2.5	$[E]$
Cell size	d_c	10	$[L]$/pixel
Cell target volume	V_0	400	$[L^2]$/pixel^2
Tissue size	$l_x \times l_y$	1280×1280	$[L]$/pixel
Tumour radius	r_0	100	$[L]$/pixel
Number of stronger cells	N_f	[0,6,12,24]	n/a

Measured physical quantities

We provide here the mathematical and/or computational definition of the physical quantities used to assess the invasion behaviour of tumours in *in silico* experiments. Quantities which are defined for both the homogeneous and heterogeneous tumour cases are derived in the homogeneous case in taking $\mu_f = \mu_b$ and/or $N_f = 0$.

- **Invasion length:** The invasion length $\Lambda(\mu_b, \mu_f, N_f, t)$ is the sum of the radial distances from the initial tumour boundary to each cancer cell i, being either a bulk or strong tumour cell, that is outside the initial tumour boundary at time t:

$$\Lambda = \sum_i (r_i(t) - r_0) \tag{3}$$

  We introduce the following notations for the invasion length in the case of homogeneous tumours, $\Lambda_b(\mu_b, t)$ and in the case of heterogeneous tumours, $\Lambda_f(\mu_b, \mu_f, N_f, t)$. Each value of Λ reported in the paper is averaged over 12 different simulation runs.

- **Invasion rate:** The invasion rate is the partial derivative with respect to time of the invasion length Λ averaged over a given time period Δt:

$$\Gamma = \langle \partial_t \Lambda \rangle_{\Delta t} \tag{4}$$

  We introduce for the following notations for the invasion rate in the homogeneous case, $\Gamma_b(\mu_b)$ and for the heterogeneous case, $\Gamma_f(\mu_b, \mu_f, N_f)$. From simulation data, the invasion rate is computed over the time interval $\Delta t = 1200$ MCS, as the mean gradient of Λ between 0 and 1200 MCS using a linear regression by the least squares method. Each value of Γ is averaged over 12 different simulation runs.

- **Normalised invasion rate per stronger cell:** The normalised invasion rate per stronger cell is measured as the percentage increase in the invasion rate per stronger cell in heterogeneous tumour:

$$\Omega(\mu_b, \mu_f) = \frac{\partial_{N_f} \Gamma_f}{\Gamma_b} \tag{5}$$

  From simulation data, the normalised invasion rate per stronger cell is computed as the ratio of the mean gradient of Γ_f as function of N_f, obtained using linear regression by the least squares method, to Γ_b. Γ_f is evaluated at 58 different locations in the (μ_b, μ_f) space, for N_f= 6, 12 and 24. For each triplet (μ_b, μ_f, N_f), Γ_f is averaged over 12 different simulation runs. $\Gamma_b(\mu_b)$ is similarly calculated from the corresponding range of μ_b values and also averaged over 12 different simulation runs.

- **Proportion of fingers led by a stronger cell:** The proportion of fingers led by a stronger cell is defined as the ratio of the number of fingers led by a stronger cell to the total number of fingers emanating from the tumour. From simulation data, fingers are detected and counted using the Dijkstra algorithm [2]. Measurements are taken on five equally spaced time points between 3500 and 4500 MCS, and averaged over 12 different simulation runs and over the range of N_f for each value in the (μ_b, μ_f) parameter space.

- **Ratio of stronger cells outside the tumour:** The ratio of stronger cells outside the tumour is defined as the ratio of the number of stronger cells such that $(r_i(t) - r_0) > r_0$ to the total number of stronger cells. Measurements are taken on five equally spaced time points between 3500 and 4500 MCS, and averaged over 12 different simulation runs and over the range of N_f, for each value in the (μ_b, μ_f) parameter space.

- **Increase in number of fingers per stronger cell:** This quantity characterises the percentage increase in the number of fingers associated with the introduction of a strong cell. It is measured for a given (μ_b, μ_f) as the mean gradient of the number of fingers in respect of N_f, normalised by the number of fingers for the corresponding homogeneous tumour (at the same μ_b). This last quantity is plotted in the top panel of Fig. 3 in supplementary materials. Measurements are taken on five equally spaced time points between 3500 and 4500 MCS, and averaged over 12 different simulation runs and over the range of N_f, for each value in the (μ_b, μ_f) parameter space. The mean gradient of the ratio is evaluated using a linear regression by the least square method.

- **Increase in average finger length:** The increase in average finger length is defined as the ratio of the length of fingers for an heterogeneous tumour, for a given $(\mu_b, \mu_f,\ N_f)$ value, to the length of fingers for an homogeneous tumour, for the same μ_b value. This last quantity is plotted in the bottom panel of Fig. 3 in supplementary materials. Finger length is defined as the shortest path from the initial tumour boundary to the tip cell of the invading structure. Measurements are taken on five equally spaced time points between 3500 and 4500 MCS, and averaged over 12 different simulation runs and over the range of N_f, for each value of (μ_b, μ_f).

- **Mean contact time of cell pairs:** The mean contact time for cells pairs is computed by monitoring the times of creation and disappearance of each cell-cell contact, considering all cell pairs of given cell types between 100 and 2000 MCS. Data are pooled over 12 different simulation runs and over the range of N_f, for each value of (μ_b, μ_f).

Additional tables and figures

**Cells motile force (μ)**			→	**$\mu = \mu_c$** →	**$\mu = \mu_s$** →
**Type of tissue**	**Type of cells**	**State of the tissue** / **Measured quantity**	**Cohesive**	**Collective**	**Individual**
Single cell	Motile cell	Mean square displacement	~0	~0	Large
Homogeneous epithelial tissue	Motile cells	Spatial correlations	Low	High	Low
		Temporal correlations	Subdiffusive	Hyperdiffusive	Diffusive
Homogeneous tumour	Bulk tumour cells	Invasion rate	~0 ①	Slow ②	Fast ③
Heterogeneous tumour	Bulk tumour cells<br>Stronger tumour cells	Invasion rate	~0 ④	Fast ⑤	Fast ⑥

Table 1: Summary table linking single cell and population level behaviours (using results from [1]) with the invasion dynamics obtained for homogeneous and heterogeneous tumours in this paper.

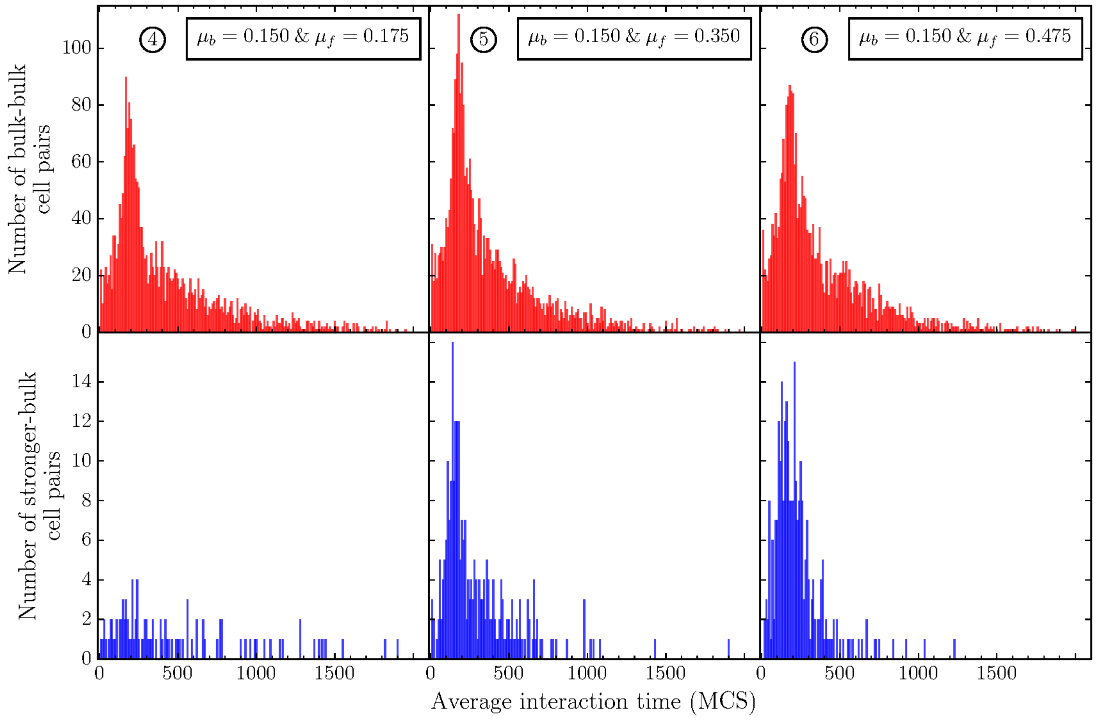

Figure 1: Histograms of cell pairs interaction times for both bulk cells pairs (upper panel) and stronger cell-bulk cell cell pairs (lower panel). Measurements for some particular μ_b and μ_f are taken every 10 MCS between 100 and 2000 MCS for each cell pair and values are pooled on 12 different simulation runs. Circled numbers relate to results in Fig2.a.

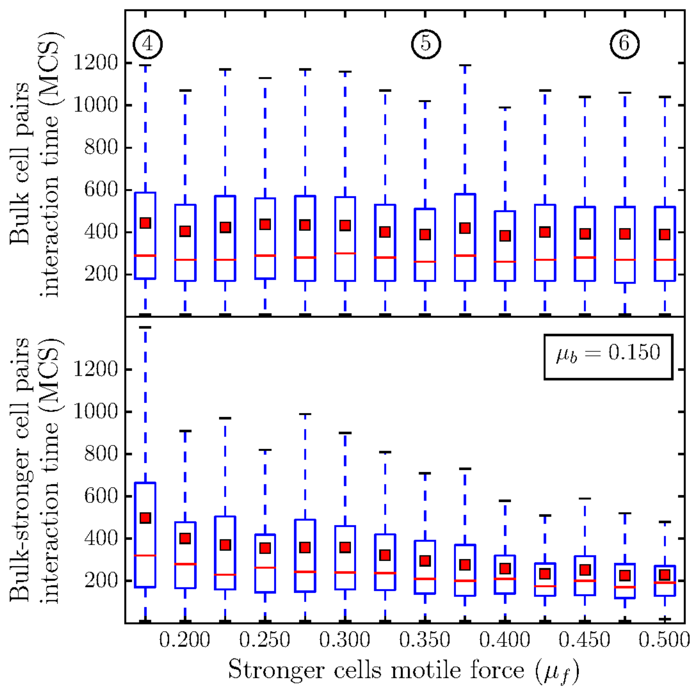

Figure 2: Box plots of cell pairs interaction times for both bulk cells pairs (upper panel) and stronger cell-bulk cell cell pairs (lower panel). Red square symbols stand for the means and red horizontal lines for the medians of the distributions. Measurements for $\mu_b = 150$ and several μ_f are taken every 10 MCS between 100 and 2000 MCS for each cell pair and values are pooled on 12 different simulation runs. Circled numbers relate to results in Fig2.a.

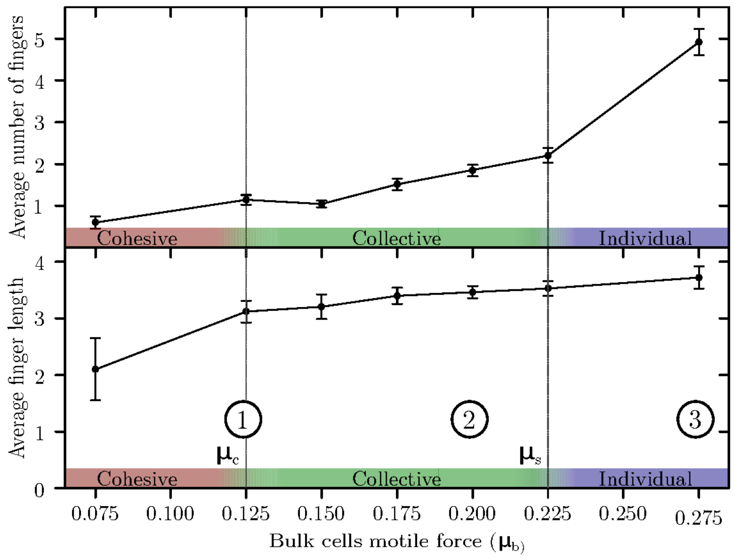

Figure 3: Morphology of collectively invading structures in homogeneous tumours. (Top panel) Average number of fingers with respect to bulk cells motile force (μ_b). (Bottom panel) Average finger length with respect to bulk cells motile force (μ_b). Measurements are taken for each numerical experiments at five equally spaced time points between 3500 and 4500 MCS, averaged over these points and then pooled over 12 different simulation runs for each value of μ_b. Error bars stands for SEM. Circled numbers relate to results in Fig1.a.

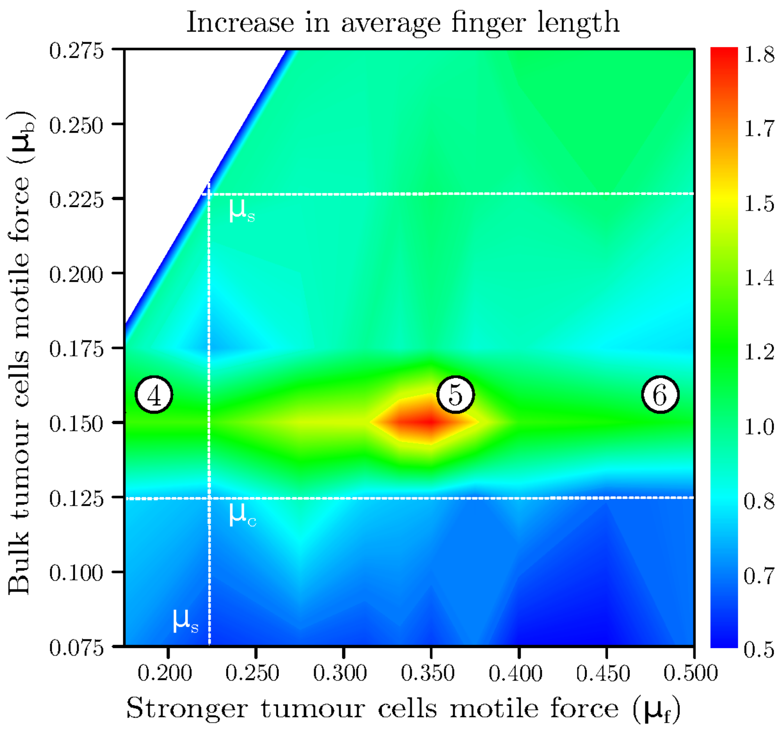

Figure 4: Heat map of the increase in average finger length with respect to μ_b and μ_f. Measurements are taken on five time points between 3500 and 4500 MCS and averaged for each point on 12 different simulation runs. Data set is the same as for Fig.2.b of the main article. Circled numbers relate to results in Fig2.a.

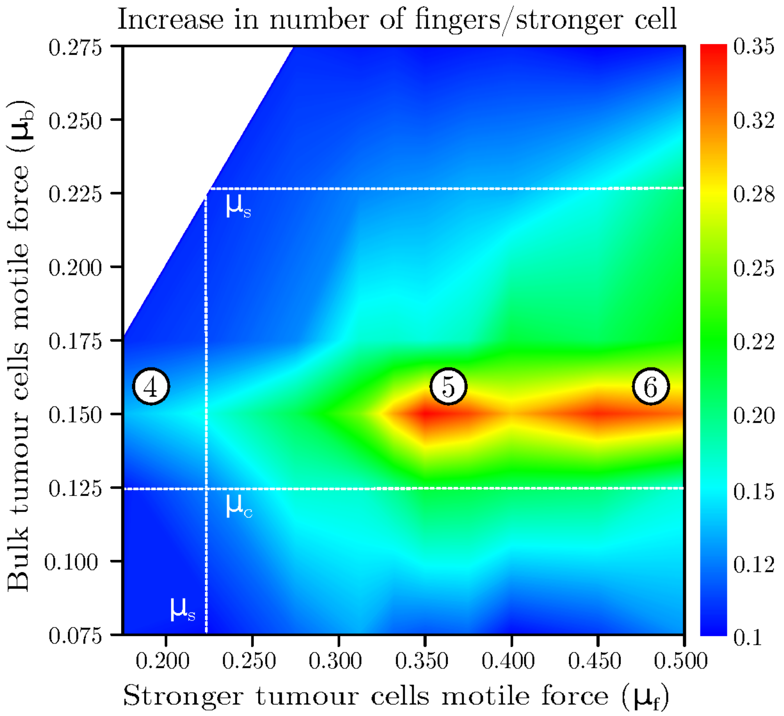

Figure 5: Heat map of the increase in number of fingers per stronger cell with respect to μ_b and μ_f. Measurements are taken on five time points between 3500 and 4500 MCS and averaged for each point on 12 different simulation runs. Data set is the same as for Fig.2.b. of the main article. Circled numbers relate to results in Fig2.a.

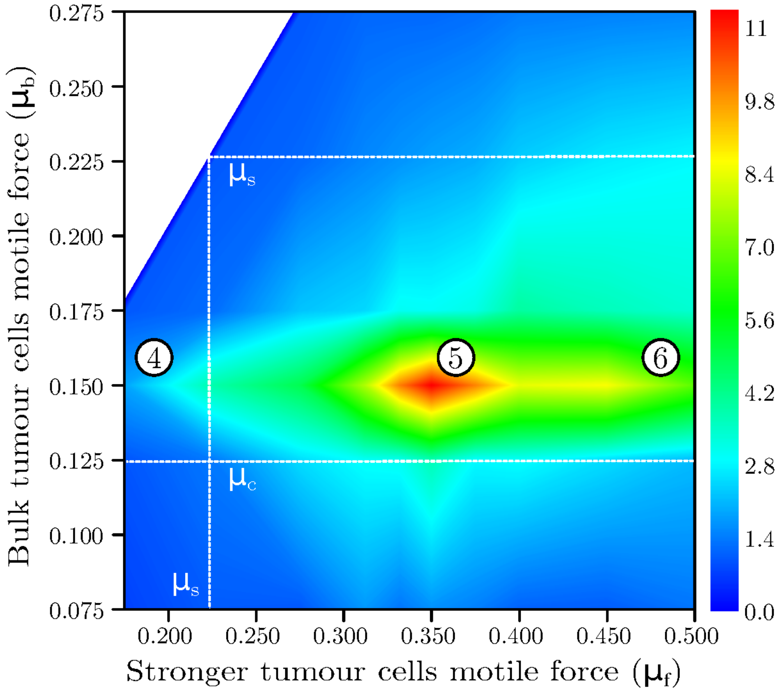

Figure 6: Heat map of the product of the increase in average finger length per the increase in number of fingers per stronger cell with respect to μ_b and μ_f. Measurements are taken on five time points between 3500 and 4500 MCS and averaged for each point on 12 different simulation runs. Data set is the same as for Fig.2.b. of the main article. Circled numbers relate to results in Fig2.a.

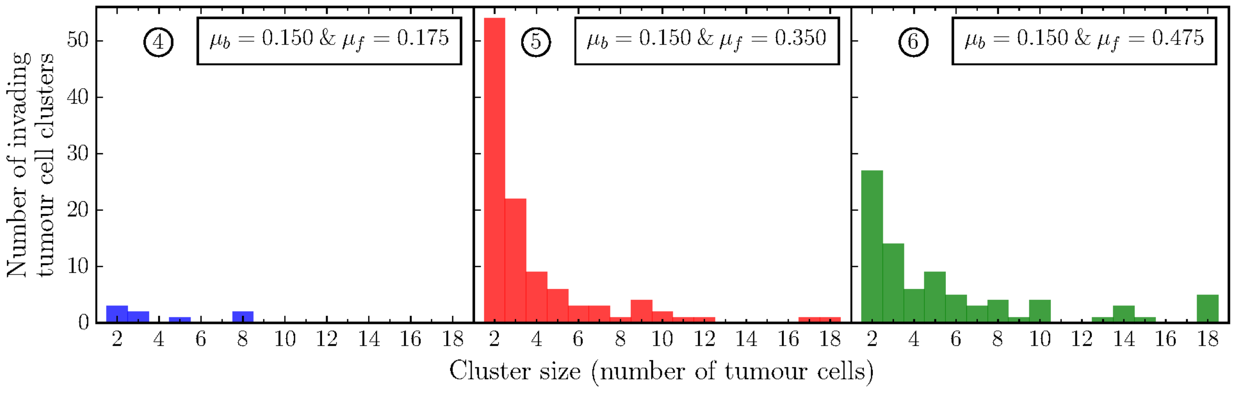

Figure 7: Histograms of invading tumour cell clusters size. Measurements for some particular μ_b and μ_f are taken at 20000 MCS and pooled for each on 12 different runs. Circled numbers relate to results in Fig2.a.

Movies of *in silico* experiments

List of all movies cited in the main article with corresponding captions. Files are available at: https://doi.org/10.17863/CAM.11252

All movies are 5000 MCS long. The frame rate corresponds to one frame every ten MCS.

**Name of the Movie**	N_f	μ_b	μ_f	**Description**
movie 1	0	0.125	—	No invasion but presence of collective cell movements in the tumour at low motile force.
movie 2	0	0.200	—	Collective invasion of bulk tumour cells at intermediate motile force ($\mu_c < \mu_b < \mu_s$).
movie 3	0	0.275	—	Tumour invasion with both collective and single cell like invasion at high motile force.
movie 4	0	0.500	—	Tumour invasion with predominance of single cell like invasion at very high motile force.
movie 5	0	0.075	—	No invasion - epithelium like dynamics at very low motile force.
movie 6	24	0.150	0.175	Low motile force. Slow collective invasion of bulk tumour cells and no stronger cell led fingers since $\mu_f < \mu_s$.
movie 7	24	0.150	0.350	Optimum of stronger cell led invasion. Both tumour cell types co-invade through long and numerous finger like protrusions with stronger cells at their leading edge.
movie 8	24	0.150	0.475	High heterogeneity in motile strength. Stronger cells invade rapidly but fail to trigger collective invasion. Bulk tumour cells invasion remains weak as a result.

[1] A.J. Kabla, Journal of The Royal Society Interface **9**, 3268(2012).

[2] E. W. Dijkstra, Numerische Mathematik **1**, 269(1959).
